# Age-related changes in consolidation of perceptual and muscle-based learning of motor skills

**DOI:** 10.3389/fnagi.2013.00083

**Published:** 2013-11-29

**Authors:** Edward F. Pace-Schott, Rebecca M. C. Spencer

**Affiliations:** ^1^Cognition and Action Laboratory, Department of Psychology, University of Massachusetts at AmherstAmherst, MA, USA; ^2^Neuroscience and Behavior Program, University of Massachusetts at AmherstAmherst, MA, USA

**Keywords:** sleep, memory, consolidation, aging, skill, motor learning

## Abstract

Improvements in motor sequence learning come about via goal-based learning of the sequence of visual stimuli and muscle-based learning of the sequence of movement responses. In young adults, consolidation of goal-based learning is observed after intervals of sleep but not following wake, whereas consolidation of muscle-based learning is greater following intervals with wake compared to sleep. While the benefit of sleep on motor sequence learning has been shown to decline with age, how sleep contributes to consolidation of goal-based vs. muscle-based learning in older adults (OA) has not been disentangled. We trained young (*n *= 62) and older (*n *= 50) adults on a motor sequence learning task and re-tested learning following 12 h intervals containing overnight sleep or daytime wake. To probe consolidation of goal-based learning of the sequence, half of the participants were re-tested in a configuration in which the stimulus sequence was the same but, due to a shift in stimulus-response mapping, the movement response sequence differed. To probe consolidation of muscle-based learning, the remaining participants were tested in a configuration in which the stimulus sequence was novel, but now the sequence of movements used for responding was unchanged. In young adults, there was a significant condition (goal-based vs. muscle-based learning) by interval (sleep vs. wake) interaction, *F*(1,58) = 6.58, *p* = 0.013: goal-based learning tended to be greater following sleep compared to wake, *t*(29) = 1.47, *p* = 0.072. Conversely, muscle-based learning was greater following wake than sleep, *t*(29) = 2.11, *p* = 0.021. Unlike young adults, this interaction was not significant in OA, *F*(1,46) = 0.04, *p* = 0.84, nor was there a main effect of interval, *F*(1,46) = 1.14, *p* = 0.29. Thus, OA do not preferentially consolidate sequence learning over wake or sleep.

## INTRODUCTION

Aging is often marked by a reduction in sleep quality; sleep efficiency decreases as wake after sleep onset increases. Rapid eye movement (REM) sleep time is reduced in older age and slow wave sleep (SWS) is reduced to an even greater extent (e.g., Ohayon et al., 2004). Such sleep changes have been posited to underlie changes in memory in conjunction with healthy aging (Buckley and Schatzberg, 2005; Hornung et al., 2005).

In young adults (YA), memory improves more over an interval containing sleep than over an equivalent interval spent awake, reflecting memory consolidation processes which are enhanced by sleep. For example, in a motor sequence learning task, a classic probe of the procedural learning system (Nissen and Bullemer, 1987), reaction time (RT) is reduced by about 18% following a 12-h interval with sleep whereas only 4% reductions are observed following a 12-h interval spent awake (Walker et al., 2002; Spencer et al., 2006). Supporting the interaction between age-related changes in sleep and memory in OA, recent evidence suggests the benefit of sleep on motor sequence learning may be reduced in OA (Spencer et al., 2007; Wilson et al., 2012). Performance on the motor sequence learning task improves by only 2% over sleep in individuals 60–80 years of age and this improvement does not differ from that observed following an equivalent interval spent awake.

Motor sequence learning is composed of learning across two dimensions: learning of the sequence of movement responses (termed motoric or muscle-based learning) and learning of the sequence of response goals (termed perceptual or goal-based learning). Willingham (1999) introduced a paradigm that may be used to dissociate the learning of these two components. In this paradigm, during a training phase, participants learned to produce a sequence of finger movements on a keyboard in response to a sequence of visual stimuli. Importantly, participants were instructed to press the key one position to the right of that indicated by the stimulus. In the later test phase, the instructions were changed such that participants were now told to press the key directly corresponding to the location of the stimulus. To probe goal-based learning, half of the participants were then shown a series of visual stimuli displayed in the same sequence as during training but, due to the changed instruction, this sequence of visual stimuli was associated with a different movement sequence. To probe muscle-based learning, the other half of the participants were shown a different sequence of stimuli that, with the changed instruction, required the same motor response as the training phase. Willingham found that participants in both groups responded faster when the cues were in the learned sequence relative to random probe blocks. Thus, he concluded that sequence learning is composed of simultaneous perceptual learning of the stimuli and muscle-based learning of the responses.

Using a variant of this paradigm, Cohen et al. (2005) sought to determine which of these components of learning – muscle-based learning, goal-based learning, or both – are consolidated over sleep. All of the young adult participants first performed the task in the training configuration. Half of the participants were trained in the evening and half in the morning. Performance was assessed 12 h later. In the second session, each of these groups was further divided such that half of the participants were tested in the configuration used to probe goal-based learning and half in the configuration used to probe muscle-based learning. Goal-based learning benefited from sleep: when the movement sequence was changed relative to encoding while the stimulus sequence was unchanged, performance selectively improved following sleep and no changes in performance were observed across the waking interval. Those participants who were probed in the muscle-based learning configuration in the second session (movement sequence unchanged; stimulus sequence changed) showed no overnight benefits but, interestingly, showed a significant increase in performance across the waking interval. Thus, the authors argue for the existence of two forms of memory consolidation, a wake-dependent process that primarily influences muscle-based learning of the movement sequence and a sleep-dependent process influencing learning of the sequence of goals (Cohen et al., 2005).

To date, studies of consolidation of motor sequence learning in OA have only examined global off-line changes in performance. Whether consolidation of goal-based learning or movement-based learning are specifically affected by aging is unknown. Thus, we used the paradigm introduced by Willingham (1999) to dissociate the consolidation of muscle-based and goal-based learning in young and OA. Given previous studies showing reduced sleep-dependent sequence learning in OA (Spencer et al., 2007; Wilson et al., 2012), we hypothesized that OA would show reduced consolidation of goal-based learning over sleep relative to YA. Changes in muscle-based learning were expected to be greater over the wake interval for YA as demonstrated by Cohen et al. (2005). However, given that wake does not change with age in the drastic fashion that sleep does, we considered the novel hypothesis that wake-dependent consolidation may be unchanged in older relative to YA.

## MATERIALS AND METHODS

### POPULATION

Participants were 62 YA (44 female) and 50 OA (37 female). YA, 18–26 years of age, participated for pay or course credit. OA, 51–80 years of age, were community dwelling, recruited via flyers and advertisements, and were paid for their time. All participants were screened against sleep and neurological disorders. Participants reported habitually sleeping greater than 5.5 h at night.

### APPARATUS

**FIGURE 1:**
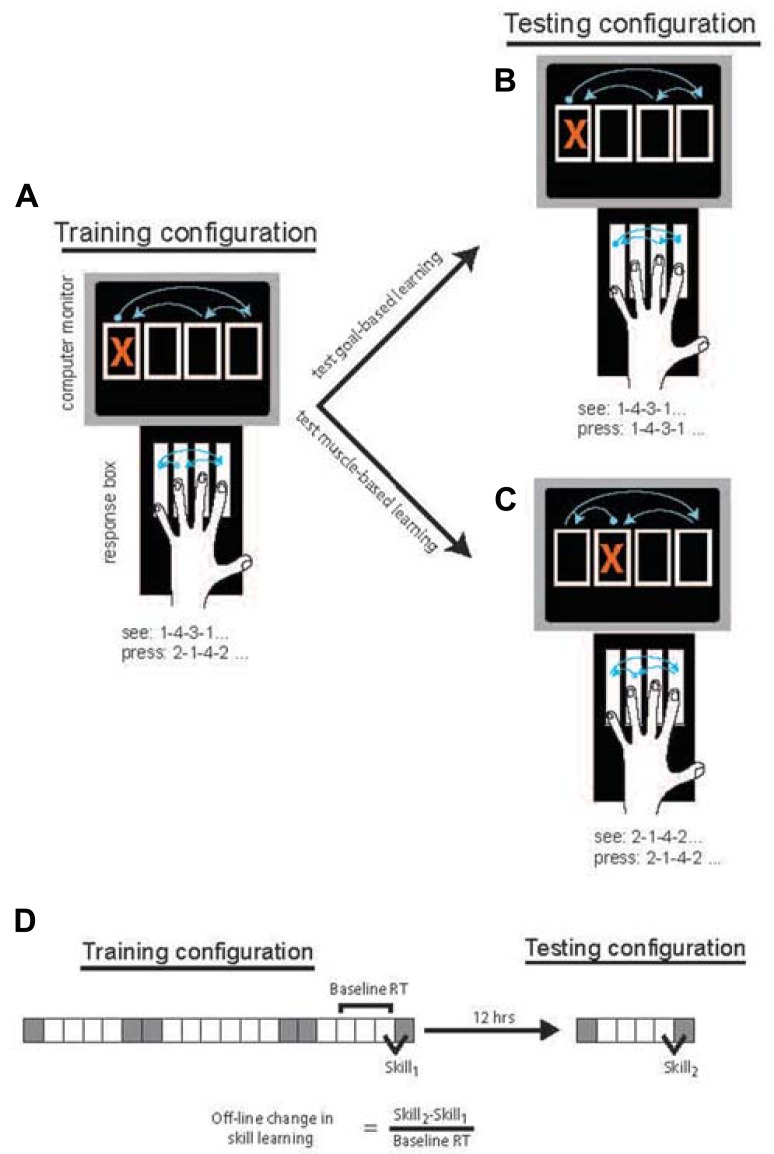
**FIGURE 1. Task design was based on Willingham (1999).** In the Training Phase **(A)**, participants responded to the location of the cue by pressing the response key one to the right of the spatially cued location (and pressing the far left key when the far right location is cued). Following 12-h either containing overnight sleep or daytime wake, participants performed the Testing Phase in which responses were made to the location directly corresponding to the cue. To probe goal-based learning **(B)**, half of the participants in each group responded to the same stimulus sequence. To probe muscle-based learning **(C)** the remaining participants responded to stimuli that were changed such that the movement response sequence was the same as during the Training Phase. The block design and measure of skill acquisition **(D)** were based on that used by Cohen et al. (2005). Gray squares = random blocks; White squares = sequence blocks.

Participants were seated at a table with a 4-key response box positioned in front of them. A computer screen displayed four vertically aligned boxes at all times. Movements were cued by the appearance of a red X in one of the four boxes (**Figures 1A–C**).

### PROCEDURE

All procedures were approved by the University of Massachusetts, Amherst Institutional Review Board and informed consent was obtained before the experiment began. OA and YA groups were further split into two groups, a Sleep group and a Wake group. These groups completed either a Goal-based learning probe or a Muscle-based learning probe in session 2. Thus, there were eight groups: 2 age groups (YA vs. OA) × 2 interval types (Sleep vs. Wake) × 2 conditions (Goal-based vs. Muscle-based) with 12–17 participants per group (see **Table 1**).

**Table 1:** Descriptive data for each group (means followed by standard error in parentheses).

	Muscle-based learning condition			
	Young adults		Older adults	
	**Sleep**	**Wake**	**Sleep**	**Wake**
Age (years)	19.8 (0.3)	19.9 (0.3)	63.5 (1.9)	62.1 (2.0)
Handedness (right:left)	14:1	16:0	10:2	12:1
PSQI	3.6 (0.6)	4.7 (0.5)	5.3 (0.8)	3.5 (0.3)
TST (min)^*^	470 (31)	478 (38)	440 (36)	437 (22)
Median RT (ms)	622 (42)	572 (18)	795 (48)	823 (51)
	Goal-based learning condition			
	Young adults		Older adults	
	Sleep	Wake	Sleep	Wake
Age (years)	20.4 (0.7)	20.2 (0.3)	61.7 (3.1)	60.7 (2.7)
Handedness (right:left)	13:1	16:1	11:2	11:1
PSQI	4.6 (0.5)	4.8 (0.5)	3.7 (0.6)	3.4 (0.6)
TST (min)^*^	424 (32)	429 (22)	466 (21)	456 (23)
Median RT (ms)	641 (29)	642 (39)	776 (43)	768 (43)

* TST is the self-reported sleep time for the experimental night (between session 1 and session 2; Sleep group) or the night prior to session 1 (Wake group).

For the Wake groups, the first session took place in the morning between 7 and 10 a.m. and the second session took place 12 h later. The Sleep groups performed the first session in the evening between 7 and 10 p.m. and the second session took place after a 12-h interval that contained overnight sleep.

All groups performed an identical Training Phase in session 1 using their non-dominant hand. The Training Phase consisted of 20 blocks. Cues were presented in a 12-item sequence on most blocks with the exception of blocks 1, 6, 7, 14, 15, and 20, which were random blocks (**Figure 1D**). In random blocks, cues were presented semi-randomly, matching frequency of each cue location to sequence blocks and constrained such that the cue could not appear in the same location on successive trials. Sequence blocks consisted of 4 repetitions of a 12-item sequence (1-4-3-1-2-4-1-3-2-3-4-2). Consistent with the method of Willingham (1999), in the Training Phase, participants were instructed to press the key to the right of the location indicated by the visual cue (**Figure 1A**). Each key was assigned one finger (**Figure 1**) and subjects were instructed to keep to this assignment. If the cue appeared in the far right location, the participant was instructed to press the key on the far left.

The second session, the Testing Phase, consisted of six blocks in which cues were randomized on blocks 1 and 6 and sequential on blocks 2–5. In this session, all participants were instructed to press the key directly corresponding to the location of the cue, again using their non-dominant hand. The cued sequence varied by condition. For the Goal-based learning condition, the sequence of cues was the same as that of the Training Phase. As such, the perceptual sequence was unchanged but the sequence of movements necessary to make those responses was changed due to the altered stimulus-response mapping (**Figure 1B**). For the Muscle-based learning condition, the stimulus sequence was shifted by one to the right (e.g., 2-1-4-2-3-1-2-4-3-4-1-3). Thus, the sequence of movements produced was unchanged relative to the Training Phase but the visual sequence of response goals was altered (**Figure 1C**).

At the beginning of both sessions participants completed the Stanford Sleepiness Scale (Hoddes et al., 1973), a subjective measure of current sleepiness. At the beginning of the first session, participants also completed the Epworth Sleepiness Scale (Johns, 1992), a subjective measure of habitual daytime sleepiness, and the Pittsburgh Sleep Quality Index (Buysse et al., 1989), a subjective measure of habitual sleep over the past 30-days. Handedness was verified with the Edinburgh Handedness Inventory (Oldfield, 1971) at the beginning of the first session. A post-experimental debriefing form (described in Spencer et al., 2007) administered at the end of session 2, was used to probe explicit awareness of the sequence.

### DATA ANALYSIS

Block structure was similar to that of Cohen et al. (2005) so that off-line changes could be similarly measured. Specifically, of interest was the amount of skill learning in session 2 relative to session 1. Skill learning was calculated as the median RT for the final random block in each session minus the median RT for the final sequential block of each session. The difference in skill learning across sessions (Skill_2_ - Skill_1_) was normalized to the average of the median RT in the final 3 sequence blocks in session 1 (**Figure 1D**). The difference in skill learning can also be thought of as the “transfer” of learning from session 1 to session 2 that is made possible by the consolidation of this learning between sessions. Given changes in stimulus-response mapping between Skill_1_ and Skill_2_, it may be argued that normalization should be to sequence block performance in session 2 where RTs were faster due to direct mapping. Notably, all effects are unchanged when normalization is based on session 2 RT, likely because of the high correlation between session 1 RT (blocks 17–20) and session 2 RT (blocks 3–5), *r* = 0.806, *p* < 0.001. This normalization procedure was performed to adjust the off-line differences to the theoretical “room for change” that may differ across individuals and, more so, age groups. Specifically, the larger RTs produced by the slower (often the older) individuals typically produced larger absolute difference values, a systemic bias that was eliminated by normalization to a baseline RT. Off-line changes were compared across groups using an ANOVA with factors Age (YA vs. OA), Interval type (Sleep vs. Wake), and Condition (Goal-based vs. Muscle-based). *Post-hoc* comparisons used unpaired *t*-tests as indicated.

## RESULTS

### GROUP DIFFERENCES

Descriptive variables for the eight groups are presented in **Table 1**. The age of the four YA groups did not differ, *F*(3,61) = 0.54, *p* = 0.65, nor did the age of the four OA groups, *F*(3,49) = 0.24, *p* = 0.87. Groups also did not differ in measures of self-reported habitual sleep (as measured by the PSQI; main effect of Age: *F*(1,104) = 1.304, *p* = 0.26; main effect of Condition: *F*(1,104) = 0.134, *p* = 0.72; main effect of Interval: *F*(1,104) = 0.172, *p* = 0.68; all interactions: *p* > 0.14) or habitual sleepiness (as measured by the Epworth Sleepiness Scale; main effect of Age: *F*(1,104) = 2.952, *p* = 0.089; main effect of Condition: *F*(1,104) = 0.658, *p* = 0.42; main effect of Interval: *F*(1,104) = 1.292, *p* = 0.26; all interactions: *p* > 0.25). Finally, subjective sleepiness at the start of each session, as measured by the Stanford Sleepiness Scale, did not differ across age groups, *F*(1,104) = 2.170, *p* = 0.14. Thus, while YA and OA groups differed in age, these groups and the Sleep/Wake subgroups did not have any other apparent baseline differences.

### ERRORS

As is often the case in the serial RT tasks, error rates were relatively low (mean accuracy across all blocks in both sessions for YA: 95.3%; mean accuracy across all blocks in both sessions for OA: 97.3%). Accuracy did not significantly differ based on Age, *F*(1,104) = 2.73, *p* = 0.10; Condition, *F*(1,104) = 1.43, *p* = 0.24; or Interval type, *F*(1,104) = 2.11, *p* = 0.15. Interactions were non-significant (all *p*’s > 0.16). Moreover, errors did not significantly increase on random blocks relative to sequence blocks consistent with instructions to “move quickly while maintaining accuracy” [session 1 main effect of block type (block 19 vs. block 20), *F*(1,103) = 0.79, *p* = 0.38; session 2 main effect of block type (block 5 vs. block 6), *F*(1,103) = 0.58, *p* = 0.45]. Given that error rate was low and differed little across groups and block type, subsequent analyses were based on correct trials only.

### SKILL ACQUISITION

Skill_1_ provides a measure of skill acquisition, prior to consolidation, that can be used to compare baseline performance across groups. A 3-way ANOVA revealed no significant main effect of age, *F*(1,104) = 2.36, *p* = 0.13, reflecting no age-related change in acquisition of this motor sequence learning task as we have reported previously (Wilson et al., 2012). The main effect of Interval type was also not significant, *F*(1,104) = 0.054, *p* = 0.82. Given that Skill_1_ was measured in the evening for the Sleep groups and in the morning for the Wake groups, the lack of an effect of Interval type on acquisition suggests that performance on this task did not vary by time-of-day (reflecting lack of circadian influences on performance). The main effect of Condition was marginally significant, *F*(1,104) = 3.50, *p* = 0.06. However, given that task requirements for Goal-based and Muscle-based learning were identical in session 1 (participants had no knowledge of how the mapping/sequences would change in the Testing Phase), this difference can only be attributable to random variation. No interactions were significant (*p*’s > 0.11).

### OFF-LINE CHANGES IN SKILL LEARNING

**FIGURE 2:**
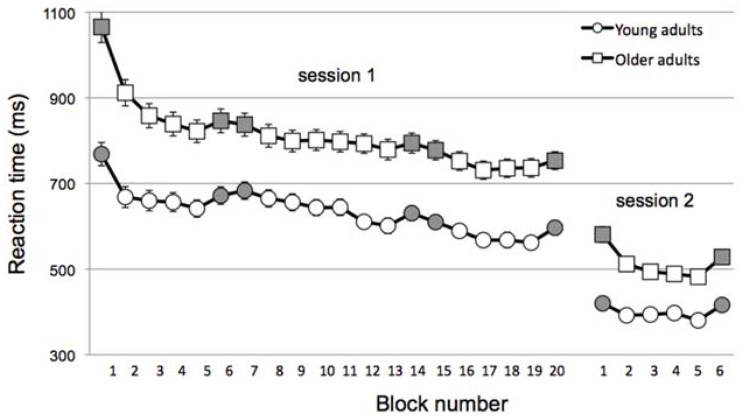
**Reaction time across blocks (gray = random; white = sequential) and sessions for young (circles) and older (squares) adults.** Note that data is collapsed for Sleep/Wake and Muscle-based/Goal-based groups within in age group for the sake of simplicity. Error bars, which are small, represent standard error.

As illustrated in **Figure 2**, OA were much slower than YA overall (mean RT across all session 1, main effect of Age, *F*(1,104) = 39.7, *p* < 0.001; mean RT across session 2, main effect of Age, *F*(1,104) = 37.2, *p* < 0.001]. Given this, the change in skill learning was normalized to RT as described above.

Not surprisingly, all groups were faster in session 2, reflecting the change to a simpler stimulus-response mapping (mean RT for session 1 blocks 16–19 vs. mean RT for session 2 blocks 2–5, main effect of Session, *F*(1,105) = 5.46, *p* = 0.02). Of interest was whether the off-line consolidation differed across groups. Indeed, a 3-way ANOVA of the inter-session change in skill showed a significant main effect of Age, *F*(1,104) = 6.1, *p* = 0.015. Moreover, the interaction of Condition × Interval was nearly significant, *F*(1,104) = 3.77, *p* = 0.055 and the 3-way interaction was significant, *F*(1,104) = 3.95, *p* = 0.049.

**FIGURE 3:**
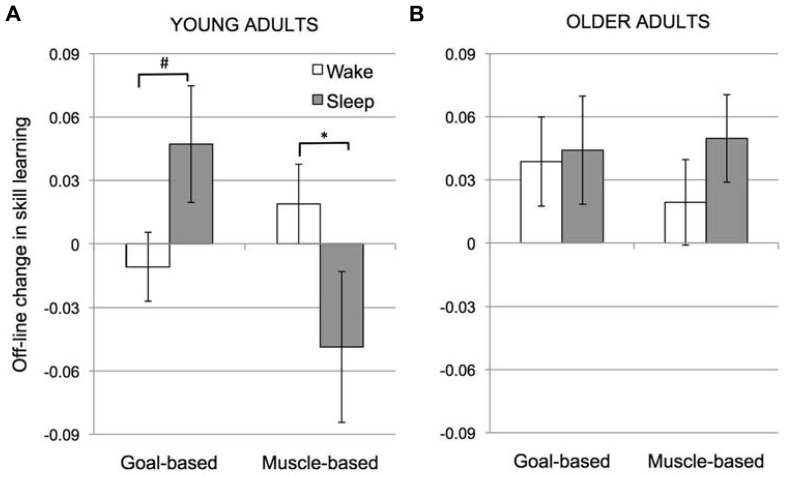
**Off-line change in skill learning (Skill**_**2**_ - Skill_**1**_adjusted to the RT at the end of session 1) for young **(A)** and older adults **(B)**. Error bars represent standard error; # marginal significance (*p* = 0.07); *significant (*p* = 0.02).

A *post-hoc*, 2-way ANOVA (Condition × Interval) for the intersession change in skill for YA only revealed no significant main effects (Condition, *F*(1,58) = 1.15, *p* = 0.29; Interval, *F*(1,58) = 0.47, *p* = 0.50]. However, the interaction of Condition × Interval was significant, *F*(1,58) = 6.58, *p* = 0.013. As seen in **Figure 3A**, in YA, transfer of learning across sessions in the Goal-based learning condition tended to be greater over sleep than wake (unpaired *t*-test: *t*(29) = 1.47, *p* = 0.072) whereas transfer in the Muscle-based learning condition was significantly greater over wake than sleep (unpaired *t*-test: *t*(29) = 2.11, *p* = 0.021). Like Cohen et al. (2005), significant improvements in goal-based learning were observed following sleep in the YA (i.e., intersession change in skill learning > 0, *t*(13) = 1.9, *p* = 0.04). Goal-based learning was unchanged over wake (intersession change in skill learning not different from 0; *t*(16) = 0.56, *p* = 0.59). Likewise, muscle-based learning was unchanged over wake (intersession change in skill learning not significantly different from 0; *t*(15) = 1.11, *p* = 0.28). Notably, here we find that muscle-based learning was significantly reduced over sleep (intersession change in skill learning < 0; *t*(14) = 2.05, *p* = 0.051).

**Figure 3B** depicts a thoroughly different pattern for the OA participants. The main effect of Condition was not significant, *F*(1,46) = 0.007, *p* = 0.93, nor was the main effect of Interval, *F*(1,46) = 1.14, *p* = 0.29. Importantly, the interaction between Condition and Interval observed in YA was not present in the OA group, *F*(1,46) = 0.04, *p* = 0.84.

### SEQUENCE AWARENESS

To query sequence awareness, a post-experiment debriefing form was completed which queried participants’ belief as to whether they had been assigned to a fictitious “sequence” vs. “random” group as well as their past musical experience, a potential determinate of finger skill (see Spencer et al., 2007). Overall, awareness of the sequence was low and did not differ between groups (main effect of Age: *F*(1,104) = 8.486, *p* = 0.09; main effect of Condition, *F*(1,104) = 0.650, *p* = 0.42; main effect of Interval, *F*(1,104) = 2.520, *p* = 0.12; all interactions: *p* > 0.45). Musical experience also did not differ between groups, χ^2^ = 6.434, *p* = 0.60.

## DISCUSSION

Here, we replicate an intriguing finding of Cohen et al. (2005) that, in YA, consolidation of goal-based learning is greater over sleep than wake whereas consolidation of muscle-based learning is greater over wake than sleep. Importantly, we demonstrate that, in OA, consolidation does not differ for intervals of sleep and wake.

### ROLE OF SLEEP AND WAKE ON OFF-LINE CONSOLIDATION IN YOUNG ADULTS

Willingham (1999) demonstrated that motor sequence acquisition occurs simultaneously in the perceptual and motor dimensions. However, a study by Cohen et al. (2005) suggests that consolidation of these may be independent given wake-dependent enhancements of muscle-based learning and sleep-dependent enhancements of goal-based learning. Consistent with this, in the present study skill learning in the Goal-based condition was greater following sleep than wake in YA. Others have demonstrated that both perceptual learning (Gais et al., 2000) and rule extraction (using a visual presentation akin to that of the present study; Wagner et al., 2004) are improved over sleep in healthy YA, a benefit associated with memory replay during sleep. Thus, coordinated replay in the hippocampus and visual cortex during sleep may underlie improvements in goal-based learning.

The Muscle-based learning condition measured the consolidation of learning of the sequence of motor responses. While the visual input changed with respect to the Training Phase, the response sequence was unchanged in the Testing Phase. Here we found a greater intersession change in skill over wake relative to sleep. This difference was driven by a decrease in muscle-based learning following an interval with sleep. Learning of the sequence of responses is thought to rely on supplementary motor area and premotor cortex (Bischoff-Grethe et al., 2004). In isolation, such learning is unlikely to benefit from hippocampal-based replay. Rather, facilitation of goal-based learning over sleep may account for the apparent reduction in muscle-based learning over sleep. Even when muscle-based learning was probed in the Testing Phase among Sleep subjects, consolidation of goal-based learning would have also occurred (participants were unaware of condition differences). Consolidating the memory of the sequence of goals over sleep may have interfered with the expression of muscle-based learning when presented with the altered sequence of goals in session 2. Conversely, when the memory of the goal sequence is not consolidated (over wake), no such interference occurs and muscle-based learning is unchanged in the Testing Phase compared to the Training Phase in this condition (**Figure 3A**). In other words, we posit that consolidation in one dimension may influence performance in the other dimension.

Given that stimulus-response mapping was simpler (direct) in session 2 compared to session 1 (press the key to the right of the cued location), the inter-session change in skill reflects improvements both due to off-line consolidation and to this change in stimulus-response mapping. As we have no reason to think that decreases in RT associated with the shift in mapping would vary by interval type (sleep vs. wake), we associate intersession changes with difference in consolidation over sleep and wake. We note that Cohen et al. (2005) avoided this confound by having participants move with their right hand in the Training Phase and with their left hand in the Testing Phase which began at the end of session 1 and the left hand was again retested in session 2. Our study was based on the design of Willingham (1999) which allowed Training and Testing performance to be measured within the same limb to avoid age-related differences in interlimb transfer of skill (Hinder et al., 2011) that would yield a confound under the Cohen et al. (2005) approach.

We also considered whether intersession changes could be explained by the shift from indirect to direct mapping between the stimulus and response across sessions. However, the change in mapping is expected to benefit the Sleep and Wake groups equally. It is important to consider alternative interpretations. First, rather than reflecting differences in sleep vs. wake on consolidation, differences between Sleep and Wake groups may reflect differences in the time of encoding or recall, in other words, a circadian influence on performance. However, counter to this interpretation, there was no difference in session 1 performance across groups in spite of the varying time of day at which they took place (Wake group in the morning; Sleep group in the evening).

### AGE-RELATED CHANGES IN OFF-LINE CONSOLIDATION

Contrary to YA, OA showed no sleep-specific gain in goal-based learning. This finding is consistent with a number of previous studies. In one such study (Wilson et al., 2012), we contrasted sleep-dependent consolidation of motor sequence learning on a simple variant of the serial RT task (Nissen and Bullemer, 1987) with over-sleep changes on a declarative, word-pair learning task. We reported that performance on the word-pair learning task was similarly greater following sleep compared to wake in both older and YA. However, consolidation of motor sequence learning was absent in the OA. Unlike YA, there was no difference in the change in RT over sleep relative to wake. Likewise, Siengsukon and Boyd (2008) found no sleep-specific changes in performance of OA who performed a continuous tracking task in which participants learned a sequence of cursor positions.

Cohen et al. (2005) posited that the wake-dependent changes they observed came about via enhanced plasticity of motor regions over wake. It is possible that a reduced benefit of wake compared to sleep in the Muscle-based learning condition in OA is the result of reduced plasticity with age (e.g., Fathi et al., 2010). Alternatively, the absence of a wake benefit on performance in OA may further reinforce the idea that off-line changes in muscle-based learning interact with off-line changes in goal-based learning in the healthy young adult. As proposed above, assuming that goal-based learning is consolidated over sleep in YA, muscle-based learning following sleep may be reduced in YA due to enhanced conflict (between the remembered stimulus and actual stimulus) relative to wake in the movement condition. In OA, if goal-based learning is not preferentially consolidated over sleep or wake, any conflict in the Muscle-based learning condition should not differ for the sleep and wake groups.

## CONCLUSION

Consistent with previous studies, these results demonstrate that motor sequence learning is not preferentially enhanced over sleep in OA as seen in YA. Novel to the present study, we suggest that this impairment is evident in the multiple levels at which a movement sequence is encoded and represented. However, the present results also suggest that consolidation of muscle-based and goal-based learning may not be completely independent, supporting the need for further research on age-related changes in consolidation.

## Conflict of Interest Statement

The authors declare that the research was conducted in the absence of any commercial or financial relationships that could be construed as a potential conflict of interest.

## AUTHOR CONTRIBUTIONS

Edward F. Pace-Schott collected and contributed to the analysis and interpretation of the data. Rebecca M. C. Spencer conceived of the project, contributed to the analysis and interpretation of the data and prepared the manuscript.
